# Complete Ureteral Avulsion

**DOI:** 10.1100/tsw.2005.13

**Published:** 2005-01-28

**Authors:** V. Gupta, T. C. Sadasukhi, K. K. Sharma, R. G. Yadav, R. Mathur, V. Tomar, S. S. Yadav, S. Priyadarshi, P. Gupta

**Affiliations:** Department of Urology, SMS Medical College & Hospital, Jaipur, Rajasthan, India

**Keywords:** Ureteral avulsion, ureteroscopy, injury

## Abstract

Complete avulsion of the ureter is one of the most serious complications of ureteroscopy. It requires open or laparoscopic intervention for repair. This case report emphasizes its management and presents recommendations for prevention in current urological practice.

## CASE REPORT

A 32-year-old male presented to us with right flank pain experienced for the last month. Pain was colicky in nature, radiating to genitalia, associated with vomiting. No history of haematuria was there. Bowel habits were normal. There was no history suggestive of any other system involvement. Examination was unremarkable. On investigation, he was found to have a right mid ureteric stone 1 cm in size.

Ureteroscopic removal was planned. Forceful placement of semirigid ureteroscope resulted in instrument drag, which hampered its maneuverability. An attempt at extraction produced ureteral avulsion, whereby about 12 cm of the ureter was brought out with the ureteroscope (Figs. 1 and 2). As the injury was recognized immediately and the patient was stable, repair was done based on standard open surgical techniques. The bladder was found to be of adequate capacity. A Boari flap was fashioned and a nonrefluxing ureteral reimplantation performed over a double J stent. The stent was removed at 6 weeks. Follow-up IVP showed prompt excretion of dye with good results (Fig. 3).

## DISCUSSION

Lyon et al.[1] predicted, "Disasters, such as perforation of the ureter, are a distinct possibility if care and thought are not practiced." Most complications of ureteroscopy are minor, necessitating only close observation or minimal intervention. Major complications of ureteroscopy, however, may have severe and lasting consequences. Complications are considered major if operative intervention is required for resolution or if the complication is life threatening. Ureteric avulsion is uncommon, but represents the most serious complication of the ureter during basket extraction of a large calculus without prior fragmentation[2]. A review of 33 published series between 1984 and 1992 comprising 5,117 patients revealed 17 avulsions[3].

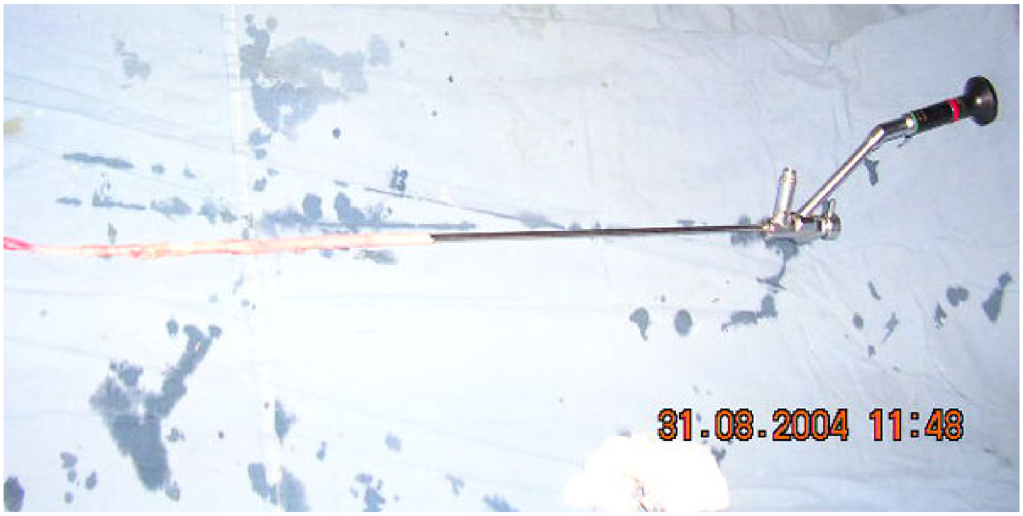

**FIGURE 1**. Ureteric avulsion.

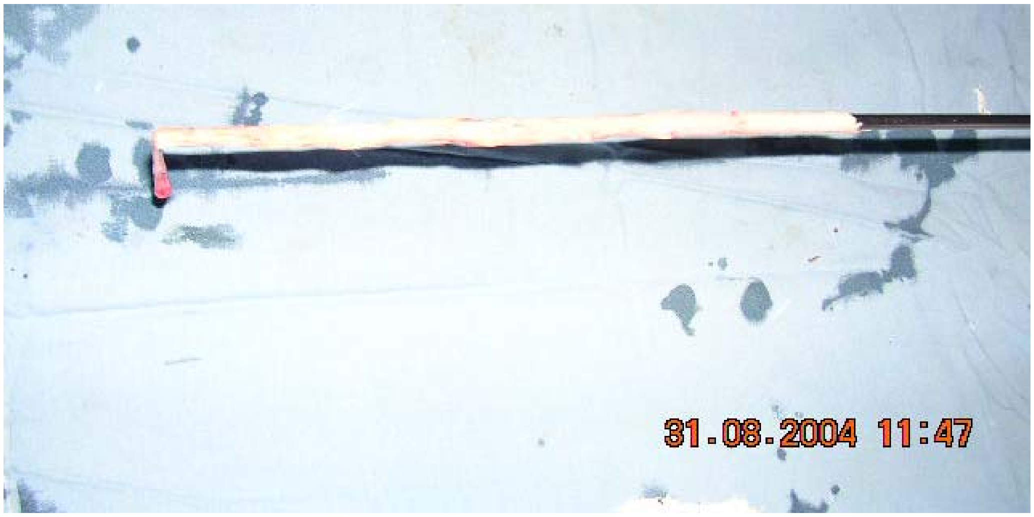

**FIGURE 2**. Complete avulsion of ureter.

In the present case, the avulsion occurred because of continued force applied to introduce the ureteroscope without prior dilation of intramural part of the ureter. Most ureteral avulsions can be managed acutely. Treatment options after complete ureteral avulsion are dependent on the length and location of viable remaining ureter as well as on the functional status of the renal unit. If the avulsion involves the distal ureter, ureteroneocystostomy is recommended. Avulsion of the middle third of the ureter may require a psoas hitch or Boari flap. Proximal ureteral injuries may be repaired primarily with end-to-end anastomosis over a ureteral stent, provided the ureter has not been devitalized. In the latter case, more extreme measures are required for repair, such as small bowel interposition or autotransplantation. To prevent the occurrence of this serious complication, utmost gentleness is required during the procedure. The intramural part of the ureter should be dilated to accommodate the ureteroscope if the ureteroscope does not move freely in and out of the ureter. A ureteroscope should not be used as a dilator and it should never be forcefully introduced.

**FIGURE 3**. Follow-up IVP.
